# Intra-cellular bacterial infections affect learning and memory capacities of an invertebrate

**DOI:** 10.1186/s12983-015-0129-6

**Published:** 2015-12-15

**Authors:** Noémie Templé, Freddie-Jeanne Richard

**Affiliations:** Laboratoire Ecologie et Biologie des interactions UMR CNRS 7267, Université de Poitiers, Bat. B8-B35; 6, rue Michel Brunet, TSA 51106, F-86022 Poitiers Cedex 9, France

**Keywords:** Feminization, Host parasite interactions, *Wolbachia*, Memory, Learning, Invertebrate

## Abstract

**Background:**

How host manipulation by parasites evolves is fascinating but challenging evolutionary question remains. Many parasites share the capacity to manipulate host behavior increasing their transmission success. However, little is known about the learning and memory impact of parasites on their host. *Wolbachia* are widespread endosymbionts and infect most insect species. These bacteria are maternally transmitted and mainly alter the reproduction of hosts with weak virulence. We tested the impact of parasites (*Wolbachia*) on their host learning and memory capacities. To address this question we trained individuals to one direction with positive reinforcement. We compared performances between individual *Wolbachia*-free, *Wolbachia* naturally and *Wolbachia* artificially infected individuals.

**Results:**

We report that in the host parasite interaction (*Armadillidium vulgare*/*Wolbachia*) naturally infected individuals *Wolbachia* or transinfected adult with *Wolbachia* are less likely to learn and memorize the correct direction with social reinforcement compared to *Wolbachia*-free individuals.

**Conclusions:**

Our results imply that *Wolbachia* impact in the central nervous system of their host altering the memory formation and maintenance. We conclude that host manipulation can affect cognitive processes decreasing host adaptation capacities.

## Background

Learning ability occurs in many taxa including both vertebrates and invertebrates. It is an adaptive trait that can provide behavioral plasticity at the individual level and can be beneficial in changing environments [1]. Invertebrates exhibit associative learning capacities in contexts such as foraging, mating, or egg-laying [1]. Some insects show complex cognitive abilities such as face-recognition learning in paper wasps [2] or concepts such as sameness and difference [3, 4]. Crustaceans, mainly crayfish and crabs, can also have complex learning and memory skills that range from simple forms of working memory to operant conditioning [5]. Elementary associative learning of direction in a T-maze has been demonstrated in terrestrial crustaceans, the isopod *Armadillidium vulgare* by using negative reinforcement [6] and the isopod *Porcellio scaber* by using positive reinforcement [7].

*Wolbachia pipientis* (hereafter *Wolbachia*) bacteria are intracellular alpha-Proteobacteria in a wide range of hosts, mainly arthropods (insects, spiders, scorpions, terrestrial isopods) and filarial nematodes [8–11]. They were first described as intracellular Rickettsia-like organisms in the mosquito [12]. Human pathogens of the Rickettsiaceae are obligate intracellular parasites that invade the central nervous system as part of a systemic infection and generally need an arthropod as an intermediate host [13]. *Wolbachia* are highly manipulative symbionts that have effects on their hosts that range from parasitism to mutualism, depending on the host [14, 15]. For example, *Wolbachia* is necessary for the reproduction and survival of filarial nematodes including *Wuchereira brancrofti* [16]. Manipulation by *Wolbachia* is multidimentional and can affect both reproductive and non-reproductive host life stages [17, 18] leading to changes in invertebrate host physiology, immunity and behavior [19]. The extended phenotype of *Wolbachia* can therefore affect the evolutionary processes of their host. *Wolbachia* transmission is vertical, and their fitness is directly linked to the fitness of their host [20]. The reproductive phenotypes obtained from *Wolbachia* are associated with reproductive parasitism (i) cytoplasmic incompatibility (the embryonic mortality amongst offspring), (ii) male killing (the killing of male embryos), (iii) parthenogenesis induction (induction of asexual daughter development), and (iiii) feminization (converting genetic males into functional females) [21]. Feminization is strongly suspected in many species but has actually been shown only in a few species [22].

Direct effects of neuroinvasion on its host remain largely unknown. A better understanding of *Wolbachia* neurological infections on their hosts can shed light on the ecological and evolutionary aspects of the infection. Because infected invertebrates by *Wolbachia* can be intermediate hosts in the infection of vertebrates, the ecological and evolutionary consequences of invertebrate infection may be of broader importance that effects of the infection on invertebrates alone.

*Wolbachia* are known to accumulate in the nervous system (brain tissues and nerve cord) of their invertebrate hosts [23–25]. In *Drosophila*, the most infected areas by *Wolbachia* are the central brain and the subesophageal ganglion brain. *Wolbachia* show specific distribution preferentially localized in self-renewing neuroblasts instead of ganglion mother cells [23], allowing a potential impact of the symbionts on their hosts’ behavior. The central brain includes the antennal lobes, which receive input from the olfactory sensory neurons, and the mushroom bodies, which provide sensory learning and memory capacities [26–28]. In terrestrial isopods, *Wolbachia* are in reproductive and non reproductive tissues including the central brain and the nerve cord [20].

In order to better understand *Wolbachia* impact on its host behavior, the terrestrial isopod *Armadillidium vulgare* can serve as a model system to investigate direct effects of intracellular parasitism of neurological tissue by *Wolbachia*. We used the pathogenic strain *w*VulC which is widespread and invasive and is considered as not locally adapted yet as the selection should favor most benign strains [20, 29]. Here, we present the first study investigated learning in *A. vulgare* according to the sex of the isopods and the presence of *Wolbachia* using naturally infected female (feminized male), *Wolbachia*-free female and *Wolbachia*-injected individuals of both sex.

## Results

**Fig. 1:**
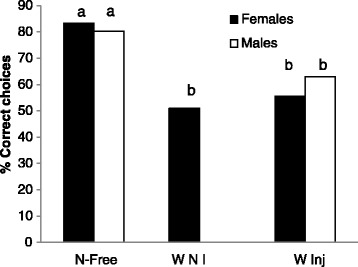
Learning performance of *A. vulgare* during training on right and left direction. Data show the results of blocks of three consecutive training visits for each individual in percentage. The letters a and b correspond to significant differences between groups. W-Free: *Wolbachia* - free individuals*;* **W NI**: *Wolbachia* naturally infected individuals; **W Inj**: *Wolbachia* injected individuals

Maze learning was not significantly different between males and females (X^2^ = 0.33; ddl = 1; p = 0.56; Fig. 1). At the same time, maze learning was not significantly different between tested individuals with natural *Wolbachia* infections and *Wolbachia*-injected specimens (X^2^ < 1.97; ddl = 1; p > 0.16; Fig. 1). However, the training success was significantly higher for *Wolbachia*-free females as compared to naturally infected and injected females (X^2^ = 27.5; p < 0.001 and X^2^ = 13.13; p = 0.0003 respectively) and for uninfected males as compared to injected males (X^2^ = 5.34; p = 0.02).

**Fig. 2:**
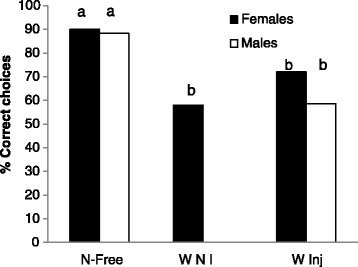
Memory performance of *A. vulgare* one hour after training. Data show the results of successful individuals in percentage. The letters a and b correspond to significant differences between groups. W-Free: *Wolbachia* free individuals*;* **W NI**: *Wolbachia* naturally infected individuals; **W Inj**: *Wolbachia* injected individuals

Individuals that had learned correct responses in the Y-apparatus during the learning phase were then used for the memory test 1 h later with no reinforcement in the apparatus. *Wolbachia*-free males and females did not significantly differ in the memory test (X^2^ = 0.14; p = 0.71; Fig. 2). Similarly, there was no difference between sexes infected with *Wolbachia*, whether naturally infected or injected (X^2^ < 1.53; p > 0.21; Fig. 2). However, the memory success was significantly higher for *Wolbachia*-free females compared with naturally infected or injected with *Wolbachia* females (X^2^ = 22; p < 0.001 and X^2^ = 5.29; p = 0.021 respectively) and for *Wolbachia*-free males compared to males injected with *Wolbachia* (X^2^ = 12.84; p = 0.0003; Fig. 2).

After behavioral tests, individuals were randomly sampled and tested for *Wolbachia* prevalence. All analysis confirmed individuals *Wolbachia* infection both in the brain and the nerve cord of naturally infected (N = 10 for each) and *Wolbachia* injected individuals (Males: *N* = 27 and *N* = 30 in the nerve cord and the brain respectively; Females: *N* = 20 and *N* = 26 for the nerve cord and brain respectively). We also confirmed the absence of *Wolbachia* detection by PCR in *Wolbachia* free individuals.

## Discussion

Our results demonstrated the capacity of an invertebrate, the terrestrial isopod *Armadillidium vulgare*, to learn turns in an experimental maze. Learning occurred in individuals of both sexes both with and without infections of the intra-cellular bacterium *Wolbachia*. Our results show, however, that the probabilities of learning and of memorization are both lower for individuals carrying *Wolbachia*. Memorization tests of individuals carrying *Wolbachia* revealed no significant difference compared to random turns.

Non-genetic mechanisms such as learned behavior can influence individual fitness and evolutionary change. It is known that the incoming sensory stimuli serve the brain in its search for the behavioral action. In *A. vulgare*, there is a strong tendency for individuals to aggregate [30]. This tendency to join other individuals can include components of learning and memory. Gregarious lifestyle is an essential requirement for social learning both in vertebrates and invertebrates [31, 32]. Moreover, hypothesis of social learning capacities have been shown in non-colonial and eusocial insects [33, 34], crustaceans [35] and in solitary vertebrates such as reptiles [36]. In our study diminished learning and memory performance of individuals carrying neural tissue *Wolbachia* infected could be due to a decrease of an individual’s stimuli-perception (intraspecific chemoreception), or it could be due to changes in the tendency to aggregate.

Short term memory is a result of changes in synaptic strength mediated by modifications of the synapse in the appropriate neurons [37]. Infection with the neurotropic parasite *Toxoplasma gondii* results in behavioral alterations such as learning and memory in rats due to accumulated changes in neurophysiology and can be the result of neural cell biology impact (neurotransmitter synthesis, signal transduction, synapse formation and dendritic arborization) of the host [38]. *Wolbachia* are present in the nervous system (brain and nerve chain) of their invertebrate hosts [23–25]. For example, in *Drosophila*, *Wolbachia* influence mate choice, mating frequency and olfactory-cues performance [39–44] and male aggression by specific strain [45]. Locomotion behaviors in response to olfactory cues are influences by *Wolbachia* depending on the host background and also the contextual environment [39, 41]. The expression of olfactory receptor neurons being related to the speed of response to a smell makes it possible to make a link between *Wolbachia* and the regulation of gene expression involved in the olfaction-receptors [46]. Moreover, olfactory processes and in particular neuromodulators plays a significant role in olfactory learning [47]. *D. melanogaster* hosting the *Wolbachia* pathogenic strain *w*MelPop, in high-density infection, present a lower gene expression of a specific neurotransmitter biosynthetic pathway decreasing the production of the neurotransmitter Octopamine which reduced male aggression [45]. No gene expression or behavioral changes were observed for the naturally infected strain *w*Mel or the benign strain *w*Mel [45]. Dopamine neurotransmitteur biosynthesis pathway involved many genes associated with phenoloxydase production and potentially with the melanization pathway. However, hemolymph extracted from mosquitoes and flies infected by *Wolbachia* (strains *w*Mel or *w*MelPop) present differences in host’s immune responses which are not due to dopamine levels [48]. In the mosquito vector of dengue *Aedes aegypti, Wolbachia* induce a range of effects including the abnormal behavioral phenotypes “shaky” and “bendy”. Even if the level of dopamine levels is higher in the heads at specific ages, again such changes alone cannot explain the phenotypes differences [49].

There are many symbionts known to modify their host behaviour and for the first time we showed direct evidence of *Wolbachia* learning impact on their host. The exact mechanisms by which *Wolbachia* alter their host behavior remains unknown in crustaceans. In other species range from mammals to invertebrate, a review highlighted the four proximate mechanisms by which symbionts impact host’s behavior by affecting the biosynthesis of neural peptides and neurotransmitters as well as the neurotransmitter receptor protein abundance, affecting the central nervous system architecture and development and finally manipulation of sex pheromones [46].

Host manipulation can lead to various behavioral changes with both proximate and ultimate consequences [50]. A parasitic virus can increase male reproductive success by intensifying their calling frequencies and sex pheromones production [51]. Parasites can also alter the perception of other individuals notably in mate choice [43, 52, 53]. Nematomorph parasites, which develop in terrestrial insects, induce their hosts to commit a suicidal jump into water and also alter the circadian activity even without being in direct contact with the host brain [54, 55]. Alteration of the behavior by parasites which reside in the central nervous system can lead to increase Gammarids host attack by predator facilitating parasite propagation [56]. Our results demonstrate for the first time that infection of the neural tissues of *A. vulgare* with the intracellular bacterium *Wolbachia* can affect the host’s learning and memory, leading to changes in the isopods’ tendency to aggregate.

## Conclusions

Host reduction of learning capacities could be an unadaptative side effect caused by the feminization potentially decreasing infected host fitness. Such behavioral alteration could potentially contribute in evolutionary strategies of avoiding or slowing down the parasites’ transmission in the population. *Wolbachia* infection of other invertebrate hosts may also affect their learning and memory capacities with potential negative impact of the host fitness link to cognition performances. Such finding opens to new approach for understanding host/parasite interactions and in particular on *Wolbachia* impact on signal interpretation, learning and long term memory consequences in various hosts.

## Methods

*Armadillidium vulgare* (Latreille, 1804) (Crustacea, Isopoda) were maintained in laboratory conditions (20 °C, natural photoperiod of Poitiers, France 46°40’N) with *ad libitum* food (fresh carrots and dried leaves of linden). Specimens of *A. vulgare* were derived from individuals collected in Helsingör, Denmark and infected by the *Wolbachia w*VulC strain [57]. Each spring, gravid females have been isolated, offspring have been sexed, and males and females separated in different plastic boxes (26 × 13 cm) before sexual maturity.

In the gregarious terrestrial isopod, *A. vugare,* and interindividual attraction occurs at short distance [58]. In their host, *Wolbachia* induce feminization of genetic male into functional female (neo-female) changing also individual phenotype (morphologically, reproductively, behaviorally). Consequently, the host sex ratio in the progenies of infected mothers leads to 70 to 80 % of female according to the transmission rate of *Wolbachia* [59] and a low proportion of males (20 % in the field) [22]. In infected lineages, all individuals that are genetically male and naturally infected are feminized by the bacteria *Wolbachia* [60].

As negative control we used naturally *Wolbachia*-free individuals originally sampled from the same location. Their negative infection status was inferred from an equilibrated sex-ratio as opposed to feminized lineages and also controlled by absence of *Wolbachia* detection by PCR on gonads.

For our experiments we used one-year old virgin individuals of similar size. All individuals were tested in di-ecdysis or beginning of pre-ecdysis (C/D_0_) [56].

**Fig. 3:**
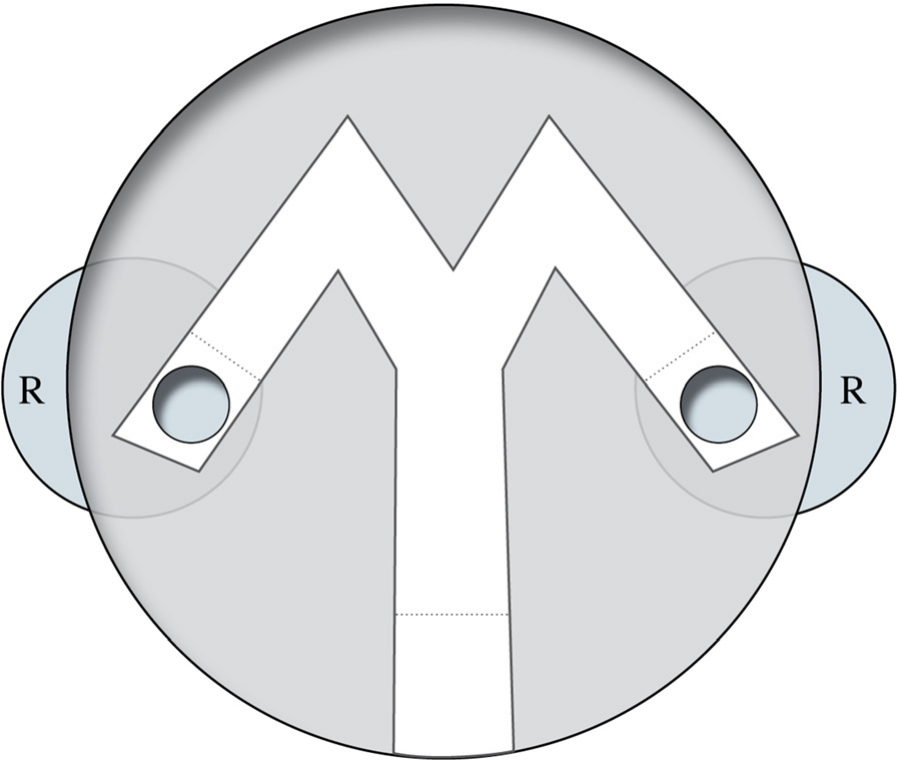
Experimental set-up used for training and retention test. R: reward chamber

Specimens were trained in Y test chamber (as described in Beauché and Richard [58]) with both arms ending with two extra chambers (“reward chamber”) down the main Petri dish (9.5 cm diameter) covered with a filter paper renewed between each experiment (Fig. 3). One of the chambers contained five individuals of the opposite sex than the tested individual. In the apparatus, tested individuals could not see the presence of the other individuals. Only chemical cues could lead to the correct direction.

Males, females, and feminized individuals were trained to choose one arm of the T-maze in order to gain access to individuals of the opposite sex. Half of the study individuals were trained to choose the left arm and the other half the right arm. If the correct arm was chosen the tested individual was allowed to stay with its conspecifics for two minutes (in the “reward chamber”). If the incorrect arm was chosen, the individual was retested with a maximum of three times. When the tested individual reached the criterion of three reward choices in a row, it was used for the memory test one hour later. We trained at least 60 of each group for the memory test (97 males, 105 *Wolbachia*-free females and 135 naturally infected females). Memory tests were performed with the identical maze set-up but without reinforcement. If individual moved to the empty reinforcement zone of the correct side, it was considered to be a positive score.

### Wolbachia injection experiments

We also conducted the same test with forty-six males and forty-seven females that had been injected with *Wolbachia*. Aposymbiotic *A. vulgare* (50 one year-old virgin females and 50 one year-old virgin males) were infected by *w*VulC. For injection, ovary suspensions were prepared with the ovaries of five *A. vulgare* symbiotically associated with *w*VulC. The ovaries were collected and crushed into 1 ml of Ringer solution. The resulting suspension was filtered through a 1.2 μm pore membrane, and 1 μL of each filtrate was injected in a small hole pierced in each individual’s cuticle, using a thin glass needle, into the posterior part of the hemocoel [61]. The delay between *Wolbachia* injection and the learning test was more than 60 days, ample time for the bacteria to infect all tissues of transinfected individuals. This protocol was applied to inject 3 independent batches of individuals. Previous study revealed no effects of *wVulC* injection for *A. vulgare* females on different life history traits [62]. Animals were then used for behavioral assays two months post-injection. As ringer injection will mimic natural injury that can occur in wild population (cause by predators) or by incidents during molt when the cuticle is soft such group wasn’t tested for behavioral tests.

#### Quantification of Wolbachia in host’s tissues

As the quantity of *Wolbachia* in the host can change across individual molt cycle, individuals were kept in the −20 °C after behavioral assay. The quantification of *Wolbachia* by quantitative PCR (qPCR) was performed on separated tissus. All the qPCR amplifications were performed with DNA sampled from the CNS (i.e., nerve chord = nerve cells and neighboring adipocytes) and the gonads. We compared 10 individuals per treatment (naturally infected female - which received the bacteria vertically from their mother, injected males and females, and *Wolbachia*-free females for control). All the qPCR reactions were performed using Roche LIGHTCYCLER 480 under the following conditions in 10 μl:5 μl of SYBR Green MasterMix (Roche), 0.5 μl of 10 μM specific primers wsp208f (5’- TGG-TGC-AGC-ATT-TAC-TCC-AG-3’) and wsp 413r (5’-TCG-CTT-GAT-AAG-CAA-AAC-CA-3’), which amplified 205 bp of a single-copy of the gene *wsp*, 3 μL of sterile water and 1 μL of DNA (between 10 and 80 ng of DNA). The thermal cycling used an initial denaturation period of 10 min at 95 °C, followed by 45 cycles of denaturing temperature at 95 °C for 10 s, the annealing temperature for the reaction was 60 °C for 10 s and 72 °C for 20 s. A melting curve (65 to 97 °C) was recorded at the end of each reaction in order to check that the PCR product was unique. Efficiency of the PCR reaction was calculated. Standard curve was plotted using 6 dilutions of *wsp* purified PCR product (wsp copies. μL^−1^: 5.8 ×10^1^, 5.8 ×10^2^ 5.8 ×10^3^, 5.8 ×10^4^, 5.8 ×10^5^, 5.8 ×10^6^). *wsp* copy number was estimated by calculation in reference to the standard curve. The total DNA quantity (i.e., host and *Wolbachia*) of each sample was used to normalize *wsp* gene copy number. The results are thus given in number of *wsp* copies by ng of total DNA. For each condition (individual*organ*Wolbachia strain), two independent technical replicates were performed.

#### Statistical analysis

We used chi-square tests to determine whether the experimentally measured choice frequencies were significantly different from random.
